# Neonatal mortality in countries of the Americas, 2000–2020: trends, inequalities, and target-setting

**DOI:** 10.26633/RPSP.2024.4

**Published:** 2024-01-22

**Authors:** Pablo Duran, Patricia Soliz, Oscar J. Mujica, Daniel A. Cueva, Suzanne J. Serruya, Antonio Sanhueza

**Affiliations:** 1 Latin American Center for Perinatology, Women’s Health, and Reproductive Health Montevideo Uruguay Latin American Center for Perinatology, Women’s Health, and Reproductive Health, Montevideo, Uruguay.; 2 Department of Evidence and Intelligence for Action in Health Pan American Health Organization Washington, D.C. United States of America Department of Evidence and Intelligence for Action in Health, Pan American Health Organization, Washington, D.C., United States of America.; 3 Independent Consultant Tarragona Spain Independent Consultant, Tarragona, Spain.

**Keywords:** Infant mortality, perinatal death, health inequities, social determinants of health, sustainable development, Americas, Mortalidad infantil, muerte perinatal, inequidades en salud, determinantes sociales de la salud, desarrollo sostenible, Américas, Mortalidade infantil, morte perinatal, iniquidades em saúde, determinantes sociais da saúde, desenvolvimento sustentável, América

## Abstract

**Objective.:**

To analyze temporal trends and inequalities in neonatal mortality between 2000 and 2020, and to set neonatal mortality targets for 2025 and 2030 in the Americas.

**Methods.:**

A descriptive ecological study was conducted using 33 countries of the Americas as units of analysis. Both the percentage change and average annual percentage change in neonatal mortality rates were estimated. Measurements of absolute and relative inequality based on adjusted regression models were used to assess cross-country social inequalities in neonatal mortality. Targets to reduce neonatal mortality and cross-country inequalities were set for 2025 and 2030.

**Results.:**

The estimated regional neonatal mortality rate was 12.0 per 1 000 live births in 2000–2004 and 7.4 per 1 000 live births in 2020, representing a percentage change of –38.3% and an average annual percentage change of –2.7%. National average annual percentage changes in neonatal mortality rates between 2000–2004 and 2020 ranged from –5.5 to 1.9 and were mostly negative. The estimated excess neonatal mortality in the 20% most socially disadvantaged countries, compared with the 20% least socially disadvantaged countries, was 17.1 and 9.8 deaths per 1 000 live births in 2000–2004 and 2020, respectively. Based on an extrapolation of recent trends, the regional neonatal mortality rate is projected to reach 7.0 and 6.6 neonatal deaths per 1 000 live births by 2025 and 2030, respectively.

**Conclusions.:**

National and regional health authorities need to strengthen their efforts to reduce persistent social inequalities in neonatal mortality both within and between countries.

## RESUMEN

**Objetivo.:**

Analizar las desigualdades en la mortalidad neonatal y las tendencias en el transcurso del tiempo entre el 2000 y el 2020, y establecer metas en materia de mortalidad neonatal para el 2025 y el 2030 en la Región de las Américas.

**Métodos.:**

Se realizó un estudio ecológico descriptivo con información de 33 países de la Región de las Américas que se usaron como unidades de análisis. Se calculó tanto la variación porcentual como la variación porcentual anual media de las tasas de mortalidad neonatal. Se utilizaron mediciones de la desigualdad absoluta y relativa basadas en modelos de regresión ajustados, para evaluar las desigualdades sociales en los diversos países en cuanto a la mortalidad neonatal. Se establecieron metas de reducción de la mortalidad neonatal y de las desigualdades en los diversos países para el 2025 y el 2030.

**Resultados.:**

La tasa de mortalidad neonatal en la Región fue de 12,0 por 1 000 nacidos vivos en el período 2000-2004 y de 7,4 por 1 000 nacidos vivos en el 2020, lo que representa una variación porcentual del –38,3% y una variación porcentual anual media del –2,7%. Las variaciones porcentuales anuales medias de las tasas de mortalidad neonatal a nivel nacional entre el período 2000-2004 y el 2020 oscilaron entre –5,5 y 1,9, y fueron en su mayor parte negativas. El exceso de mortalidad neonatal estimado en el 20% de los países más desfavorecidos socialmente, en comparación con el 20% de los países menos desfavorecidos socialmente, fue de 17,1 muertes por 1 000 nacidos vivos en el período 2000-2004 y de 9,8 muertes por 1 000 nacidos vivos en el 2020. Al extrapolar las tendencias más recientes, se prevé que la tasa de mortalidad neonatal de la Región alcance valores de 7,0 y 6,6 muertes neonatales por 1 000 nacidos vivos en el 2025 y el 2030, respectivamente.

**Conclusiones.:**

Las autoridades de salud nacionales y regionales deben fortalecer las medidas para reducir las desigualdades sociales que aún persisten en materia de mortalidad neonatal, tanto entre los distintos países como dentro de cada país.

## RESUMO

**Objetivo.:**

Analisar as tendências temporais e desigualdades em mortalidade neonatal entre 2000 e 2020 e estabelecer metas de mortalidade neonatal para 2025 e 2030 na Região das Américas.

**Métodos.:**

Estudo ecológico descritivo examinando 33 países das Américas como unidades de análise. Foram estimadas a variação percentual e a variação percentual anual média das taxas de mortalidade neonatal. Foram usadas medidas de desigualdade absoluta e relativa baseadas em modelos de regressão ajustados para avaliar desigualdades sociais entre países em termos de mortalidade neonatal. Foram definidas metas de redução da mortalidade neonatal e das desigualdades entre países para 2025 e 2030.

**Resultados.:**

A taxa regional estimada de mortalidade neonatal foi de 12,0 por mil nascidos vivos em 2000–2004, e de 7,4 por mil nascidos vivos em 2020, representando uma variação percentual de -38,3%, e uma variação percentual anual média de -2,7%. As variações percentuais anuais médias nacionais das taxas de mortalidade neonatal entre 2000–2004 e 2020 variaram entre -5,5 e 1,9 e, em sua maioria, foram negativas. O excesso estimado de mortalidade neonatal nos países que estavam entre os 20% mais desfavorecidos socialmente, em comparação com os países entre os 20% menos desfavorecidos, foi de 17,1 e 9,8 mortes por mil nascidos vivos em 2000–2004 e 2020, respectivamente. Com base em extrapolação das tendências recentes, estima-se que a taxa de mortalidade neonatal regional deve atingir 7,0 e 6,6 mortes neonatais por mil nascidos vivos em 2025 e 2030, respectivamente.

**Conclusões.:**

As autoridades de saúde nacionais e regionais precisam intensificar seus esforços para reduzir desigualdades sociais persistentes na mortalidade neonatal, tanto dentro dos países quanto entre eles.

Significant reductions in child mortality, deaths in children younger than 5 years which is regarded as a core indicator of population health and well-being, have been observed over the past few decades in the Region of the Americas (1). However, risks of neonatal mortality, deaths occurring in the first 28 days of life, remain high. Neonatal death is the main component of child mortality and contributes substantially to the burden of mortality in the region (2, 3). For instance, in 2020, 47% of all deaths in children younger than 5 years around the world were neonatal deaths, but in Latin America and the Caribbean, this figure was 56% and in North America it was 54% (2).

Along with the substantial burden of neonatal mortality, the high contribution of preventable causes of deaths and social inequalities are major challenges. Reducing preventable neonatal mortality and the large differences in neonatal mortality between groups (e.g., based on ethnicity, sex, place of residence, and socioeconomic status) within countries have been widely discussed in the literature (4–6). Just over 30 years have passed since the Convention on the Rights of the Child was adopted (7), and the importance of strengthening the response to neonatal mortality from a perspective of human rights and equality is more pressing than ever.

Countries have sustained their commitment to advance towards the elimination of deaths from preventable causes within the framework of the Sustainable Development Goals (SDGs) (8) and other specific frameworks and strategies (9). For instance, the SDG target for the neonatal mortality rate (NMR), SDG indicator 3.2.2, is fewer than 12 neonatal deaths per 1 000 live births in each country by 2030 (10). Furthermore, ending preventable neonatal deaths and expanding enabling environments to achieve this goal are some of the objectives of the *Plan of action for women’s, children’s, and adolescents’ health 2018–2030* (11) and the *Global strategy for women’s, children’s, and adolescents’ health (2016–2030)* (12).

This paper presents a descriptive ecological study of 33 countries of the Americas with the purpose of: (i) analyzing regional NMR trends between 2000 and 2020; (ii) exploring ecosocial inequalities in neonatal mortality between countries; and (iii) defining neonatal mortality reduction scenarios in order to set targets specifically focused on reducing geographical inequalities.

## METHODS

We conducted a descriptive ecological study. The units of analysis were the 33 countries of the region of the Americas with a population exceeding 90 000 inhabitants in 2020 for which the United Nations Inter-Agency Group for the Estimation of Childhood Mortality produces neonatal mortality estimates (2). This was the source for NMR estimates (2). Five periods were used to study trends: four 5-year periods from 2000 to 2019 (2000–2004, 2005–2009, 2010–2014, and 2015–2019) and 2020, which marked the beginning of the coronavirus disease 2019 (COVID-19) pandemic. Mortality rates for each 5-year period were computed by calculating the average (weighted by number of live births) annual rates during the 5-year period. To analyze NMR trends during the periods considered, both the percentage change and average annual percentage change were estimated.

The Sustainable Development Index (SDI) was used to rank countries from the most to the least socially disadvantaged (13). The SDI is a summary metric that captures three sustainable development dimensions (economic, social, and environmental) from three proxy indicators: per capita gross domestic product at constant international prices; mean years of schooling in the population older than 25 years; and the percentage of the population with access to at least basic sanitation services (13). The SDI assumes a unique value for each country-year ranging from 0 (least sustainable development) to 1 (highest sustainable development), estimated using data from the Institute of Health Metrics and Evaluation. For the analysis of inequalities between countries of the Americas, neonatal mortality was compared between the most and the least socially disadvantaged country. This is known as an ecosocial approach because the units of analysis are the countries (ecological study).

Gap and gradient inequality metrics were calculated. The absolute gap was calculated by subtracting neonatal mortality rates for the first and fifth quintiles of the SDI (14). The slope index of inequality (SII) and relative index of inequality (RII) were computed by fitting a negative binomial regression models. The outcome variable was the number of neonatal deaths (ND) in each country in the population at risk (live births [LB]), and the independent variable was the ridit value or fractional rank for each country, which represents the relative social position of each country within a range of 0 to 1 (14). More specifically, the following model was adjusted: *log(ND) = a + b Ridit + log(LB)*, where *log(·)* is the natural logarithm and from which the equivalent equation *ND = LB × exp(a + b Ridit)* was obtained, where *exp(·)* is the exponential function. In this model, when *Ridit = 0* (country with poorest social conditions), ND = LB *× exp(a)* and when *Ridit = 1* (country with optimal social conditions), ND = LB *× exp(a + b).* By calculating the difference between these two values, the SII is produced, and by calculating the ratio between these values, the RII is obtained, which are expressed as: *SII = [exp(a) – exp(a + b)] × 1000* and *RII = exp(a)/exp(a + b)*. The *a* and *b* parameters were estimated by adjusting a negative binomial regression model using the maximum likelihood method.

Finally, targets were set to reduce both the regional NMR and geographical inequalities between countries by 2025 and 2030 based on a methodology to jointly improve average rates and reduce geographical inequalities in SDG 3 indicators (15). This method consists of five steps: (i) calculate the regional average annual percentage change for the NMR; (ii) define geographical strata according to NMR values at the baseline; (iii) apply a proportional progressivity criterion to the average annual percentage change for each stratum to set larger reductions in NMR to geographical strata with higher NMR values compared to strata with lower NMR values; (iv) set average regional and national NMR targets; and (v) establish targets to reduce geographical inequalities (15). In total, four geographical strata were defined by taking into account: (i) the SDG 3 NMR target of 12 neonatal deaths per 1 000 live births; (ii) the regional NMR target of 9 neonatal deaths per 1 000 live births by 2030 established in the *Sustainable Health Agenda for the Americas 2018–2030* (16); and (iii) a maximum allowable threshold, equivalent to twice the 2030 health agenda target for this indicator (18 per 1 000 live births). This resulted in the following four strata: stratum 1, countries with NMR greater than or equal to 18 neonatal deaths per 1 000 live births; stratum 2, countries with NMR between 12 and 18 per 1 000 live births; stratum 3, countries with NMR between 9 and 12 per 1 000 live births; and stratum 4, countries with NMR lower than 9 neonatal deaths per 1 000 live births. The average annual percentage change to set the targets in the four strata was obtained from the regional average annual percentage change between the 2010–2014 and 2015–2019 5-year periods and a proportional progressivity factor of 50%. The estimated regional average annual percentage change in NMR between 2010–2014 and 2015–2019 was –1.7% – a value similar to that estimated between the 2015–2019 5-year period and the year 2020. Thus, the average annual percentage change values to set NMR targets were –2.6%, –2.1%, –1.3%, and –0.9%, respectively for each of the four strata.

## RESULTS

The regional NMR for the 2000–2020 period (average) was 9.6 neonatal deaths per 1 000 live births, with a rate of 12.0 per 1 000 live births in 2000–2004 and 7.4 per 1 000 live births in 2020, representing a percentage change and average annual percentage change of –38.3% and –2.7%, respectively. The five countries with the highest NMR in the region in 2020, exceeding 12 neonatal deaths per 1 000 live births, were (ordered from highest to lowest NMR) Haiti, Dominican Republic, Guyana, Venezuela (Bolivarian Republic of), and Bolivia (Plurinational State of), whereas the five countries with the lowest rates were (ordered from lowest to highest NMR) Cuba, Canada, United States of America, Antigua and Barbuda, and Uruguay (Table 1).

### Neonatal mortality trends

**FIGURE 1.**
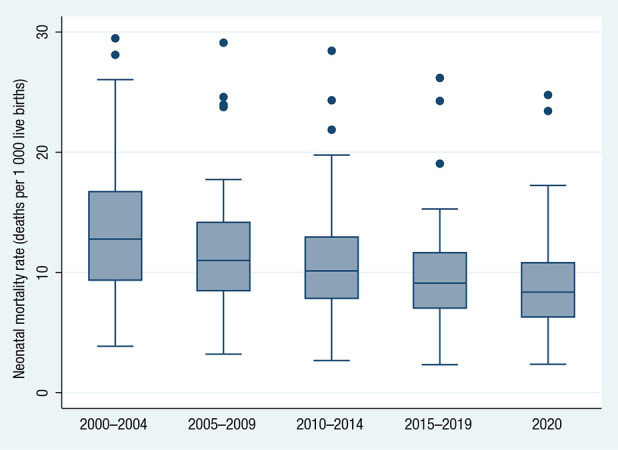
Neonatal mortality rates in 33 countries of the Americas by 5-year periods between 2000 and 2019 and in 2020

***Source*:** prepared by authors based on the results.

Figure 1 shows the distribution of NMR in the Americas over the time periods assessed. Regional mortality rates have declined, but a large disparity still exists between countries in each of the 5-year periods and in 2020.

The estimated regional NMR in 2000–2004 and 2020 was 12.0 and 7.4 neonatal deaths per 1 000 live births, respectively (Table 2). The greatest regional reduction in NMR was at the beginning of the study period, between 2000–2004 and 2005–2009 (average annual percentage change –3.9%) compared with the 2010–2014 and 2015–2019 periods (average annual percentage change –1.7%) (Table 2). Reductions in the regional NMR continued from 2015–2019 to 2020 (average annual percentage change –1.6%).

The five countries with the largest decrease in NMR between 2000–2004 and 2020 (ordered from largest to smallest reduction) were Antigua and Barbuda, Argentina, El Salvador, Peru and Bolivia (Plurinational State of), with an average annual percentage change of up to –5.5% (Table 1). Conversely, during these two periods, NMR increased in the Dominican Republic, Grenada, Saint Lucia, and Venezuela (Bolivarian Republic of), with an average annual percentage change of up to 1.9% (Table 1). Between 2015–2019 and 2020, a decrease was observed in most countries, except for Cuba, Grenada, Saint Lucia, and Venezuela (Bolivarian Republic of) whose rates were practically unchanged.

**TABLE 1.** Neonatal mortality rates in countries of the Americas across 5-year periods and in 2020, and the average annual percentage changes between the first 5-year period and 2020

Country	Neonatal mortality rate, number of deaths per 1 000 live births					AAPC
	2000–2004	2005–2009	2010–2014	2015–2019	2020	2000–2004 vs 2020
Americas (all countries)	12.0	9.9	8.7	8.0	7.4	–2.7
Antigua and Barbuda	9.3	7.2	5.2	3.9	3.4	–5.5
Argentina	10.6	8.4	7.4	6.1	4.6	–4.6
Bahamas	8.3	8.6	7.8	7.2	6.6	–1.3
Barbados	9.5	10.1	9.8	8.9	8.2	–0.8
Belize	11.1	8.7	10.1	9.1	7.7	–2.0
Bolivia (Plurinational State of)	28.1	24.6	19.8	15.3	13.5	–4.1
Brazil	16.8	12.9	10.4	9.4	8.7	–3.7
Canada	3.9	3.8	3.7	3.4	3.2	–1.1
Chile	5.4	5.4	5.3	4.8	4.4	–1.2
Colombia	12.7	11.0	9.3	7.9	7.2	–3.2
Costa Rica	7.4	6.9	6.5	6.0	5.6	–1.5
Cuba	4.0	3.2	2.7	2.3	2.4	–2.9
Dominican Republic	23.2	23.8	24.3	24.3	23.4	0.1
Ecuador	12.8	10.2	8.2	7.0	6.7	–3.5
El Salvador	13.5	10.6	8.6	7.0	6.2	–4.3
Grenada	8.1	8.3	9.8	10.9	11.2	1.8
Guatemala	20.2	17.7	15.0	12.4	11.1	–3.3
Guyana	26.0	24.0	21.9	19.1	17.2	–2.3
Haiti	29.5	29.1	28.4	26.2	24.8	–1.0
Honduras	16.6	14.2	11.8	9.8	8.8	–3.5
Jamaica	16.1	13.9	12.2	10.3	9.3	–3.0
Mexico	12.4	9.4	8.7	8.5	8.4	–2.2
Nicaragua	15.7	14.0	13.0	10.9	9.4	–2.9
Panama	14.0	12.2	10.5	8.9	8.0	–3.1
Paraguay	17.3	15.2	13.1	11.2	10.0	–3.0
Peru	14.4	11.2	9.0	7.5	6.7	–4.2
Saint Lucia	11.8	12.5	12.8	12.5	13.1	0.6
Saint Vincent and the Grenadines	13.1	13.0	11.8	9.7	9.1	–2.1
Suriname	17.0	16.1	13.9	12.0	10.9	–2.5
Trinidad and Tobago	17.5	15.7	13.6	11.7	10.6	–2.8
United States of America	4.6	4.3	4.1	3.7	3.4	–1.7
Uruguay	8.2	6.1	5.0	4.4	4.1	–3.8
Venezuela (Bolivarian Republic of)	10.4	10.3	11.1	14.6	14.6	1.9

AAPC, average annual percentage change.

***Source*:** prepared by authors based on the results.

### Social inequalities in NMR

**FIGURE 2.**
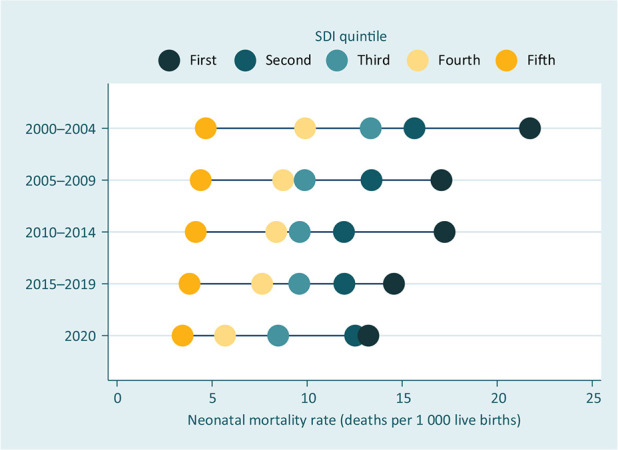
Neonatal mortality rates in 33 countries of the Americas across the Sustainable Development Index quintiles by 5-year periods and in 2020

***Source*:** prepared by authors based on the results.

The distributions of NMR in the Americas across the gradient of SDI quintiles for each of the four 5-year periods and for 2020 are shown in Figure 2. While absolute inequalities fell steadily throughout the 5-year periods, they persisted through to 2020. The estimated excess neonatal mortality in the 20% most socially disadvantaged countries, compared with the 20% least socially disadvantaged countries (according to SDI), was 17.1 and 9.8 deaths per 1 000 live births in 2000–2004 and 2020, respectively (Table 2). In relative terms, the risk of neonatal death in children in the 20% most socially disadvantaged countries in the Americas ranged from 3.8 to 4.7 times the risk in the 20% least disadvantaged countries during the periods studied. The reductions in absolute gap and relative gap between the first two 5-year periods (2000–2004 and 2005–2009) were greater than those between the 2010–2014 and 2015–2019 periods (Table 2).

The estimated SII in 2000–2004 and 2020 indicates that during these periods the excess burden of neonatal deaths was 22.5 and 12.7 neonatal deaths per 1 000 live births, respectively, to the detriment of the countries of the Americas with the lowest SDI values (Table 2). In relative terms, the estimated RII over the same periods indicates that the risk of neonatal mortality in the country with the lowest SDI value was 6.3 and 5.5 times higher than the risk in the country with the highest SDI value, respectively (Table 2). Again, greater reductions were observed in absolute and relative inequalities between the 2000–2004 and 2005–2009 5-year periods than between 2010–2014 and 2015–2019. The SII value decreased slightly between the 2015–2019 5-year period and 2020; however, this did not occur with the RII, which increased during this period.

### Setting target NMR for 2025 and 2030 in the Americas

Table 3 presents the projected NMR for countries for the years 2025 and 2030, taking the NMR in 2015–2019 as the baseline and a sustained average annual reduction of –1.7%. Based on these estimates by country, the projected regional NMRs for 2025 and 2030 (weighted average) are 7.0 and 6.6 neonatal deaths per 1 000 births, representing the regional NMR target for these 2 years (Table 3). Additionally, from the estimated NMR values for each country in the baseline period (2015–2019) and for the years 2025 and 2030, the absolute geographical gap and the relative geographical gap were estimated (Table 3). The absolute geographical gap and the relative geographical gap correspond to the arithmetic difference and ratio, respectively, in NMR between strata 1 and 4. In relative terms, an 18% decrease in the regional NMR by 2030 could be set as a target, with a 32% decrease in the absolute geographical gap and an 18% decrease in the relative geographical gap.

## DISCUSSION

This study focused on analyzing regional trends in neonatal mortality and ecosocial inequalities in 33 countries of the Americas, and estimating NMR targets for 2025 and 2030, with a view to reducing geographical inequalities between countries. The aim was to define future scenarios of neonatal mortality reduction and to contribute to the formulation of interventions that would also decrease inequality in neonatal mortality. It is important to focus efforts on improving the rate of decline in NMR over time, especially among the most socially vulnerable segments of the populations within each country.

**TABLE 2.** Neonatal mortality rates and inequality gaps by time period in the Americas

Metric	Neonatal mortality rate	Absolute gap	Relative gap	Slope index of inequality	Relative index of inequality
**Period**				
P1 = 2000–2004	12.0	17.1	4.7	22.5	6.3
P2 = 2005–2009	9.9	12.7	3.9	17.4	5.8
P3 = 2010–2014	8.7	13.1	4.2	14.0	4.9
P4 = 2015–2019	8.0	10.8	3.8	13.2	5.1
P5 = Year 2020	7.4	9.8	3.8	12.7	5.5
**Percentage change**				
P1 vs P2	–17.6	–25.7	–16.7	–22.5	–8.4
P3 vs P4	–8.0	–17.8	–8.2	–5.6	4.0
P4 vs P5	–7.8	–9.1	0.4	–3.6	7.4
**Average annual percentage change**				
P1 vs P2	–3.9	–6.0	–3.6	–5.1	–1.8
P3 vs P4	–1.7	–3.9	–1.7	–1.2	0.8
P4 vs P5	–1.6	–1.9	0.1	–0.7	1.4

***Source*:** prepared by authors based on the results.

In 2020, in six of the 33 countries in this study, the NMR was greater than or equal to 12 neonatal deaths per 1 000 live births (SDG 3 target for NMR by 2030). If these countries could reduce their NMR at an average annual percentage change of –2.6%, only two of them would reach the SDG 3 target. Countries in serious danger of failing to reach this target by 2030 are Haiti, the Dominican Republic, and Guyana, which would need to reduce their NMR at an average annual percentage change of –7.3%, –6.7%, and –3.6%, respectively, between 2020 and 2030. At the same time, the *Strategic plan of the Pan American Health Organization 2020–2025* (17) set a regional target of 6.9 neonatal deaths per 1 000 live births by 2025, taking the regional NMR in 2017 as the baseline value. However, given the findings of this study, only a third of the countries are expected to reach this target by 2025 (Table 3). This finding underscores the urgent need to promote access to maternal and neonatal health services and interventions to reduce national NMR with an equity perspective.

The *Sustainable health agenda for the Americas 2018–2030* also sets a target to “reduce the neonatal mortality rate to less than 9 deaths per 1 000 live births in all population groups, including those most at risk (indigenous, Afro-descendent, Roma, and rural populations, among others, as applicable in each country)" (16). This target is much more ambitious than previous ones on NMR, as it explicitly sets a figure for the most vulnerable populations within countries by 2030. With the predicted country NMR for 2030 in this paper, only about 40% of countries are expected to reach this target at the national level by 2030. Evidence suggests, however, that NMR in subpopulations (e.g., wealth or education quintiles.) or geographical regions (e.g., subnational levels) within countries may be higher than the national rates (6). In this regard, the proposal to set regional NMR targets for 2025 and 2030 with a focus on inequalities is relevant both from a regional and national perspective. The targets presented, based on the method used, simultaneously consider both the decrease in the regional NMR and in inequalities between countries, which is appropriate for accountability purposes. Setting explicit NMR targets using the methodology presented in this study would allow strategies to be developed that would help countries reach the 2030 neonatal mortality targets, while explicitly incorporating an equity approach.

Social inequalities in the NMR are considerable; for example, the NMR tends to be higher in the most vulnerable population groups (e.g., in the United States, preterm birth rates are higher in minority and low-income groups). This highlights the urgent need for policy-makers, governments, international organizations, and nongovernmental organizations to collaborate to end preventable neonatal deaths and address social inequalities. Furthermore, investments in health information systems and concerted efforts to improve the availability, quality, and robustness of neonatal mortality data, especially at subnational levels and in specific population groups within countries, are required. This would ensure greater accuracy and timeliness to monitor neonatal survival rates in the countries of the Americas.

**TABLE 3.** Neonatal mortality rate by country and geographical gaps for the 2015–2019 5-year baseline period and 2025 and 2030 target years

Country	Neonatal mortality rate, per 1 000 live births
		2015–2019	2025	2030
Americas (all countries)	8.0	7.0	6.6
Haiti	26.2	22.1	19.9
Dominican Republic	24.3	20.5	18.4
Guyana	19.1	16.1	14.5
Bolivia (Plurinational State of)	15.3	13.1	11.9
Venezuela (Bolivarian Republic of)	14.6	12.5	11.4
Saint Lucia	12.5	10.7	9.7
Guatemala	12.4	10.7	9.7
Suriname	12.0	10.3	9.3
Trinidad and Tobago	11.7	10.0	9.1
Paraguay	11.2	9.9	9.2
Grenada	10.9	9.7	9.0
Nicaragua	10.9	9.6	8.9
Jamaica	10.3	9.2	8.5
Honduras	9.8	8.7	8.1
Saint Vincent and the Grenadines	9.7	8.6	8.0
Brazil	9.4	8.3	7.7
Belize	9.1	8.1	7.5
Barbados	8.9	7.9	7.3
Panama	8.9	7.9	7.3
Mexico	8.5	7.6	7.0
Colombia	7.9	7.2	6.7
Peru	7.5	6.8	6.3
Bahamas	7.2	6.5	6.1
Ecuador	7.0	6.3	5.9
El Salvador	7.0	6.3	5.9
Argentina	6.1	5.5	5.2
Costa Rica	6.0	5.5	5.1
Chile	4.8	4.4	4.1
Uruguay	4.4	4.0	3.8
Antigua and Barbuda	3.9	3.5	3.3
United States of America	3.7	3.4	3.2
Canada	3.4	3.1	2.9
Cuba	2.3	2.1	2.0
**Gaps**			
Absolute geographical gap	20.2	16.0	13.7
Relative geographical gap	5.1	4.5	4.2

***Source*:** prepared by authors based on the results.

The COVID-19 pandemic caused major setbacks with regard to many SDG targets. Yet our study found no evidence to suggest significant changes in NMR regional trends or in cross-country ecosocial inequalities in 2020. Since the neonatal mortality data used in the analysis come from the estimates of the UN Inter-Agency Group for the Estimation of Childhood Mortality up to 2020 (2), it is important to acknowledge that there may be limitations in the neonatal mortality data from the countries that were used to inform these estimates and that they may differ from the data reported by countries. Our study has several limitations, starting with its ecological nature and lower degree of data granularity (level of detail). Yet the data provide an accurate picture of NMR trends at the regional level, as well as the pattern of inequality in NMR between countries. Indeed, a strength of our study is the computing of ecosocial inequalities by adjusting the negative binomial regression model. This strength notwithstanding, another limitation of our study is that it did not explore within-country inequalities, as this would have required additional data that were not available. Moreover, our proposal of goal-setting for reduction in cross-country NMR inequality does not take into account the social dimensions of inequality, only its geographical ones. Although geographical settings within a country are invariably associated with particular socioeconomic conditions, it would be desirable to explicitly identify these social dimensions of inequality to improve our ability to act on the social determinants of neonatal mortality and survival in those settings.

Given the lack of updated neonatal mortality data from 2021 onwards, the real neonatal mortality figures for this period are unknown. This lack of data is of particular concern for the most vulnerable population groups within countries; newborn babies and children in more vulnerable households may require greater protection and more interventions than other children to avoid unnecessary loss of life. It is expected that during the post-COVID-19 recovery, efforts will be strengthened to recover losses and accelerate the implementation of evidence-based and cost-effective interventions to reduce neonatal mortality. International organizations and the countries of the Americas have shown great commitment to comprehensive vaccination of the population to reduce preventable deaths from COVID-19. The same commitment is needed to end all preventable neonatal deaths that devastate millions of families year after year.

## Disclaimer.

The authors hold sole responsibility for the views expressed in the manuscript, which may not necessarily reflect the opinion or policy of the *Revista Panamericana de Salud Pública / Pan American Journal of Public Health* and/or those of the Pan American Health Organization and the World Health Organization.
